# Chicago Medical Society

**Published:** 1888-09

**Authors:** 


### SOCIETY REPORTS.

### CHICAGO MEDICAL SOCIETY.

Regular Meeting, May 7, 1888.

The President, J. H. Etheridge, M. D., in the chair.

Dr. J. L. Gray presented a specimen of gunshot wound with rupture of the heart. The points of entrance and exit were quite small. The point of entrance was between the fifth and sixth ribs; the rupture was upward. The ball passed through the body and was taken out between the tenth and eleventh ribs. It was not at all marked—no scratches on it.

The integument was not much lacerated. A peculiar point about the injury to the pericardium was that it was so small.

Dr. Frank Cary: A short time ago I read a case reported by Dr. Wescott, of a patient in the insane asylum who took a grain of atropia.

I distinctly remember a somewhat similar case which occurred at St. Luke's Hospital a few years ago. A woman about 24 years of age was acting in the capacity of assistant nurse, and one morning went to the medicine chest ostensibly to take some cough sirup, but she took down the sulphate of atropia bottle, a four-ounce vial which contained a solution of two grains atropia sulphate to the ounce. A patient who was in the room said she saw her take two teaspoonfuls. A few moments after she had taken the medicine and gone to work it was noticed that she was staggering about the ward. The nurse had her placed in bed, and the interne was summoned. He gave her ten or twelve grains of tannic acid. The pupils were widely dilated and the patient nearly unconscious when he was called into the ward. She took the poison at eight o'clock in the morning, and the interne saw her at nine. He gave her, also, in less than fifteen minutes, one-eighth grain morphine hypodermically, with a grain and a half of tartar emetic; fifteen minutes later he gave her another one-eighth grain of morphine, and one hour later one-fourth grain. The breathing at this time was quite noisy—there was a blowing expiratory sound. At this time he gave two ounces of the sulphate of magnesia. At 10.30 I saw the patient. The corners of the mouth were drawn down; she had a pinched expression, and there was a peculiar flush of the whole face. Morphine was given in doses of one-eighth to one-fourth grain every two hours from ten o'clock until two. At 2.30 the patient vomited slightly; the respirations were very shallow, from eight to ten a minute. Shaking her produced no effect whatever, but by wetting a towel in .cold water and slapping her it was found she would take deep inspirations. At this time hypodermics of brandy were given every few minutes. At five o'clock the respirations were shallow and the pulse 120. At 6 o'clock p. m. the pupils first reacted slightly to the hypodermics of morphine. At 7.30 the patient was completely paralyzed, the teeth chattered, and she had those peculiar tetanic convulsions which in the lower animals follow poisonous doses. The catheter was passsd several times during the day and small amounts of urine were withdrawn. During the night the catheter was passed without any result. At eleven o'clock the patient again vomited a small amount; the pupils were somwhat contracted. About midnight the respirations again became very shallow. Morphine was given and it was found necessary to use the battery for some time. Forty-eight hours after the ingestion of the atropine she was able to resume her duties.

Dr. F. TALIAFERRO reported a case of Œdema of the Glottis, as follows:

Mat. Daley, aged 22, of 353 South May street, Chicago, was taken with a chill, at 2 p. m., March 17, 1888, while on duty at the Grand Trunk depot, but did not leave for home till 5 p. m. Did not eat any supper. His mother gave him a hot drink and a hot mustard foot-bath and he went to bed. About 12 p. m. he vomited some greenish material. About 8 a. m. commenced to have some difficulty in breathing, and half an hour later he complained of some pain on the right side of his larynx in addition to the dyspnœa. Leeches were ordered to front of larynx about 9.15 a. m. At 10.30 a. m. a laryngeal examination not being satisfactory, the index finger revealed a stiff and swollen epiglottis. Dr. Taliaferro went to his office to prepare for tracheotomy, which he felt quite sure would be necessary. When he again reached the house he found the patient in the next room stretched out on a lounge, with his eyes staring, pupils dilated, countenance livid, and totally unconscious, looking as if each gasp would be his last. As there was no time to delay he cut as quickly as he could down upon the trachea, not considering the black and red blood which welled up successively through the incision, fixed the trachea with his left hand and cut into it and enlarged the opening and succeeded in getting the tube in without difficulty. Soon his pupils began to contract, gradually his eyelids began to close, and shortly afterwards he moved his head. Of course a good deal of blood flowed into the windpipe, which he coughed out, and this continued all the afternoon and evening.

Quite a high reaction followed his return to consciousness, his fever going up to 103°, and his pulse to 120. He gradually continued to get better until Wednesday afternoon when the tube was removed. The flesh wound healed rapidly, and in two days after removal he was not breathing through the wound at all.

Dr. J. L. GRAY desired to ask the members of the society whether it has been their experience that unusually large doses of bromides and chloral can be given in delirium tremens? At the Detention Hospital there are many cases of delirium tremens, and they take two or three times as large doses of bromide and chloral as other patients would take, apparently without any bad effect either as to the circulation or to the heart.

The PRESIDENT: With regard to the treatment of delirium tremens I wish to say that for ten or twelve years I have treated it, from the start, with chloral, the idea being to put a man to sleep as quickly as possible. I have no hesitancy in giving thirty grains every hour until the patient goes to sleep. I watch the heart very closely until he does go to sleep. I received the idea from a physician in the hospital of Edinburgh. He said that until they commenced the chloral treatment of delirium tremens they had from three to ten beds occupied with these men, but now it was their custom to get the men out in forty-eight hours, and back to work. They commenced at once with large doses, giving whatever was necessary in the way of cathartics.

Dr. H. H. FROTHINGHAM reported a case of administration of chloral in delirium tremens, which was given between eight in the evening and six in the morning, half an ounce of chloral and an ounce of bromide of potassium. Two days afterward he was admitted to the County Hospital, and while the nurse was preparing some medicine for him, he suddenly fell over on the bed and began snoring, and expired without having had any medicine whatever. On post-mortem examination it was learned that he had small hæmorrhages at the base of the brain, due, probably, to his chronic alcoholism.

Dr. L. L. MCARTHUR desired to put a case on record. A patient was brought into the hospital suffering with sciatica, which he had endured for five or six years, in spite of every variety of treatment known to medical science, except stretching the nerve. He consented to have the nerve stretched. Proper preparations for the operation were made the night before, putting the patient in the best possible condition. At the operation the buttocks were shaved and scrubbed with a carbolized solution. A clean-cut incision was made over the course of the nerve, beginning at the margin of the gluteus maximus; the nerve was readily found, exposed, and stretched, and the wound was sealed with the strictest antiseptic precautions. It was so simple and clean cut a wound, and such careful precautions were taken to get primary union, that little or no doubt was anticipated as to the early and favorable healing of the wound. In fact, the patient was promised that he would do as several other cases in the hospital had done before, probably have the wound healed up in four days, and at the end of a week be able to sit up. He was operated on at ten in the morning and by six in the evening had a temperature of 101, which rose during the night to 102½ and in the morning was 103; the next day it remained between 103 and 102½, being kept from rising higher by the influence of antipyrin. Evident sepsis being present, the wound was opened to its full extent. It had united partially by primary union; no pus was found in the wound, but the margin of the muscle had a gangrenous appearance. No drainage-tube had been introduced into the wound, but what little drainage was necessary was provided for at the lower angle of the wound by two or three strands of Seabury & Johnson's catgut. After being opened the wound was washed out with 2½ per cent. solution of carbolic acid, and packed with iodoform gauze, which, moreover, had been rubbed in iodoform; still his temperature did not drop, and in another twenty-four hours the patient died from heart-failure.

Few cases of nerve-stretching have resulted fatally. The operation is usually considered a simple one, not at all dangerous, and it is important that the unsuccessful ones be reported so they can serve as a guide.

He took the catgut, which we thought could be the only source of infection, that had been used for drainage and attempted to make cultures. He tried peptone broth, gelatine, and agar-agar mediums, but in none of them was any growth obtained, showing that it was aseptic, at least that part which remained in the body.

There is much of an outcry now against catgut, and it is being done away with largely; Volckmann will not use it.

